# Performance Assessment of Non-Gaussian Control Systems Based on Mixture Correntropy

**DOI:** 10.3390/e21111069

**Published:** 2019-10-31

**Authors:** Jinfang Zhang, Di Wu

**Affiliations:** School of Control and Computer Engineering, North China Electric Power University, Beijing 102206, China; wudify@126.com

**Keywords:** control system performance assessment, non-Gaussian, mixture correntropy, minimum entropy, EDA algorithm

## Abstract

The performance assessment of any control system plays a key role in industrial control systems. To meet the real-time requirements of modern control systems, a quick and accurate evaluation of the performance of a system is necessary. In this paper, a performance assessment method of a non-Gaussian control system based on mixture correntropy is proposed for non-Gaussian stochastic systems. Mixture correntropy can solve the problem of minimum entropy translation invariance. When the expected output of a system is unavailable, mixture correntropy combined with the estimation of distribution algorithm (EDA) is used for system identification and noise distribution estimation so as to calculate the benchmark of entropy-based performance assessment. When the expected output of a system is available, the mixture correntropy is directly used as the index to evaluate the performance of the system. To improve the real-time aspect of the performance assessment, an improved EDA is presented to obtain the evaluation index more quickly. For both Gaussian and non-Gaussian systems, the mixture correntropy and the improved identification algorithm are used for system performance assessment, and the results are compared with the minimum entropy index and the probability density function (PDF) curve coincident area index. The comparisons verify the rationality and effectiveness of the correntropy index and the rapidity of the improved EDA algorithm.

## 1. Introduction

With the rapid development of communications, microelectronics, and computer technology, industrial control systems are constantly changing, and the level of automation is constantly increasing. Fast transmission of information, rapid sampling, and control of field devices, rapid display and operation of the host computer, and increasingly higher requirements for the real-time performance of a control system are being introduced for increasingly greater numbers of industrial control systems [1]. The real-time performance of a control system is the key to the performance of the entire control system, directly affecting the control quality of the system. Therefore, it is of practical engineering significance to be able to assess the performance of a system quickly. To assess the performance of a control system, the system data are needed, and for processing a large amount of data, in addition to hardware upgrade, the speed and real-time performance can be raised by improving the algorithms used.

More attention is being paid to the performance of control systems. Continuous control system performance assessment methods have been developed and successfully applied in actual industrial production processes, but most of these methods choose variance as a performance index, and the noise is assumed to obey a Gaussian distribution. The theory of minimum variance control is combined with the performance assessment method by Harris [2]. For the first time, the concept of performance assessment index is proposed. The minimum variance is chosen as the benchmark and compared with the actual variance of the output. We use the name of the researcher Harris to name the index. This approach has a milestone impact on the future of the system performance assessment research and provides researchers with a good base. In recent years, the minimum variance control and the minimum variance index have been applied to the field of artificial intelligence [3]. However, in the actual production process, the noise disturbances of a system are often random and can be subject to any random distribution. Even if the noise of the system obeys a Gaussian distribution, the distribution of the system may be non-Gaussian because the nonlinearity of a system may occur with the system running. Therefore, the traditional Gaussian-based modeling and control method cannot meet the requirements of a stochastic distribution control system, which may lead to the results of performance assessment to deviate from the correct ones [4]. For the above two cases, the use of the mean and variance as performance indices may cause major errors.

Researchers have proposed a new method that uses a minimum entropy control strategy to assess system performance. Entropy can describe the uncertainty of a system, which is widely used in stochastic systems but rarely studied in control system performance assessment. This method could be used to evaluate the performance of Gaussian systems as well as that of non-Gaussian systems [5].

For Gaussian systems, minimizing entropy is equivalent to minimizing variance. For non-Gaussian systems, when the probability density function (PDF) of the system output is measurable, the B-spline basis function is used to approximate the output PDF [6,7,8]. The B-spline decoupling model is then converted into input and output forms. The historical output PDFs and the control input of the current time are used to obtain the output PDF of the current time. With the PDF, entropy can be determined. The premise of using the B-spline is that the output PDF can be measured [9,10]. When the output PDF is unmeasurable, the system model is represented by a controlled auto-regressive moving average (CARMA) model. With the PDF of the input noise [11], the output PDF could be obtained with the application of the probability theory. As such, whether the output PDF is measurable or not, it could converge to the desired value or the PDF of the error obeys a high and sharp distribution at 0 with controllers designed by minimizing the entropy [4], and the system performance can be evaluated with the minimum entropy.

A randomly distributed control algorithm based on the minimum entropy criterion is proposed by Zhang and Chu [12], which inspires later researchers in the field of system performance evaluation. A feedback control system performance evaluation method based on the minimum entropy criterion is proposed by Jiang [13], which is similar to the minimum variance benchmark. The key aspect of the minimum variance benchmark based assessment method is obtaining the variance of system feedback invariants, whereas the method proposed by Jiang finds the entropy value of the feedback invariants, and the entropy value is used as the benchmark of system evaluation. Zhou [5] modifies the calculation method of minimum entropy proposed by Jiang [13] and provides a detailed calculation method for the minimum entropy for discrete and continuous disturbances. The entropy value of feedback invariants is also obtained, and the entropy value is used as the benchmark for system evaluation. The problem of a minimum Shannon entropy [14] control system is analyzed by Zhang and Zhou [15]. The definition of Shannon entropy does not satisfy the “consistency,” that is, when entropy can be calculated using different methods, the results must be the same. To solve the question, a new entropy function, rational entropy (RE), is proposed and used as the performance index of the minimum RE control for general stochastic distribution control systems. The problem of nonconvex optimization is solved by the method of mean constraint. However, in this work, only the calculation method of the theoretical reference value is provided; the estimation method of the benchmark is not mentioned. Combined with rational entropy, a control system performance evaluation index based on the minimum error entropy is established by Zhou [5], which is used in a general feedback control system with non-Gaussian interference. However, the index also needs to be combined with constraints on the mean to avoid the translation invariance of entropy value.

The key to the performance assessment of a control system based on minimum entropy is to obtain the benchmark entropy, but most of the methods for obtaining the benchmark entropy are very complex. For example, when the benchmark entropy is calculated using the estimation of distribution algorithm (EDA), 1000 PDFs of the error need to be obtained in only one iteration, and the entropy values of these PDFs are calculated [5], this will need about a few minutes. When the method is applied to an actual system, the amount of data is larger. This complex calculation requires considerable time to obtain a system performance evaluation index, and cannot meet the real-time requirements of industrial control systems. Therefore, the benchmark entropy should be obtained quickly and easily, and the results of the system performance evaluation should be quickly and accurately provided.

Correntropy [16,17,18] is a measure of similarity in the kernel space. The larger the correntropy between two sequences, the smaller the difference between them. Due to the characteristics of correntropy, it is mainly used in the fields of linear regression, adaptive filtering, state estimation, identification, principal component analysis, pattern matching, and deep learning. Correntropy can suppress large outliers in signal processing and machine learning. The introduction of correntropy provides a good solution to the control and filtering of non-Gaussian systems. Mixture correntropy [19] can be considered as a generalized form of correntropy. Mixture correntropy can flexibly adjust the kernel width and weight coefficient to improve the accuracy of the correntropy. Inspired by the use of correntropy in the filter design of non-Gaussian systems, mixture correntropy is adopted to assess the performance of a control system. Compared with rational entropy, mixture correntropy calculations are simpler, the problem of the minimum entropy translation invariance can be solved.

In order to satisfy the real-time demands of modern control systems, improve the accuracy and rapidity of the evaluation indicators, mixture correntropy is chosen for performance assessment, which is a more accurate and rapid evaluation index for the non-Gaussian random distribution control system, whether the output distribution of the system is known or unknown.

We focus on the case where the expected output distribution of the system is available and unavailable, and mixture correntropy is used for the performance assessment of the non-Gaussian control system. The system model is outlined first in the next section. The third part introduces the minimum variance and minimum entropy indices of performance assessment. Due to the limitations of these two indices, the mixture correntropy is adopted. In using the mixture correntropy based index for performance evaluation, improved EDA is presented to improve the accuracy and rapidity of the method. The fourth part selects and introduces the system performance assessment indices for the cases that the expected output distribution is unavailable and available. Finally, the proposed index and improved EDA are used in a numerical simulation to verify the validity and accuracy.

## 2. Feedback Control System

**Figure 1:**
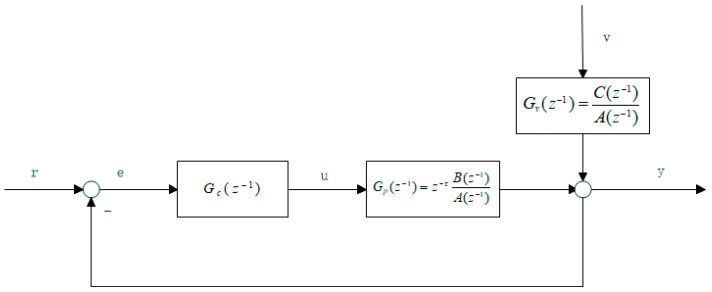
Feedback control system.

To evaluate the system performance, a feedback control system is chosen first, which is shown in Figure 1, where *r* is the setpoint; *u* is the control input; *v* is the white noise; *τ* is the system delay; and *G_c_, G_p_*, and *G_v_* are transfer functions of the feedback controller, the controlled object, and the disturbance channel, respectively.

The system is represented with a CARMA model as follows:

(1) $$A(z^{-1})y(k)=B(z^{-1})u(k-d)+C(z^{-1})v(k)$$

where *A*, *B*, and *C* could be expressed as:

(2) $$\begin{array}{l} A(z^{-1})=1+a_{1}z^{-1}+a_{2}z^{-2}+\cdot \cdot \cdot +a_{na}z^{-na}\\ B(z^{-1})=b_{1}z^{-1}+b_{2}z^{-2}+\cdot \cdot \cdot +b_{nb}z^{-nb}\\ C(z^{-1})=1+c_{1}z^{-1}+c_{2}z^{-2}+\cdot \cdot \cdot +c_{nc}z^{-nc} \end{array}$$

When *C*(*z*^−1^) = 1, the noise e(*k*) is white noise, and when C(*z*^−1^) ≠ 1, the noise e(*k*) is colored noise, i.e.,

(3) $$e(k)=C(z^{-1})v(k)=v(k)+c_{1}v(k-1)+c_{2}v(k-2)+\cdot \cdot \cdot +c_{nc}v(k-n_{c})$$

In minimum variance control and performance assessment, the estimated values of the above parameters *a_n_*, *b_n_*, and *c_n_* are obtained through model identification, and the premise is that the delay and order of the system are known.

## 3. Control System Performance Assessment Indicators

With the given system, a performance evaluation index should be selected for performance assessment of the system. At present, two main methods are used in system performance assessment. For Gaussian systems, most of the evaluation indicators are based on the minimum variance, whereas for non-Gaussian systems, the minimum entropy index is used to evaluate the performance. Since the minimum variance and the minimum entropy are equivalent in a Gaussian system, the minimum entropy could also be used to evaluate the performance of a Gaussian system.

### 3.1. Minimum Variance and Minimum Entropy Index

Assuming *r* = 0 for the system in Figure 1, the output of the system can be expressed as:

(4) $$y_{t}=\frac{G_{v}}{1+G_{p}G_{c}}v_{t}=\frac{G_{v}}{1+z^{-\tau }{\tilde{G}}_{p}G_{c}}v_{t}$$

where *v*_t_ is the estimated noise distribution, $\tilde{G}$*_p_* is the transfer function without delay, and *G_v_* is the transfer function of the disturbance which could be expressed by the Diophantine equation as follows:

(5) $$G(q^{-1})=F(q^{-1})+q^{-\tau }R(q^{-1})$$

where *F*(*q*^−1^) = 1 + *n*_1_*q*^−^^1^ + … + *n_τ_ −*_1_*q*^−^^(*τ*^^−^^1)^, the coefficients of *F*(*q*^−1^) are the impulse response coefficients of *G_l_*, and *R*(*q^−1^*) is the proper transfer function, satisfying the rest of the Diophantine identities. Substituting Equation (5) into Equation (4), Equation (4) can be rewritten as

(6) $$y_{t}=Fv_{t}+Lv_{t-\tau }$$

where:

(7) $$L=\frac{R-F{\tilde{G}}_{p}G_{c}}{1+q^{-\tau }{\tilde{G}}_{p}G_{c}}$$

*F* is independent of the manipulated variable, which is feedback invariant and can be obtained using the Diophantine equation; *L* is dependent on the controller. Therefore, when the structure and parameters of the controller are properly selected, *L* can be made zero.

In the minimum variance control, the minimum value of the system output variance is obtained by designing the controller:

(8) $$Var(y_{t})=Var(F\nu_{t})$$

With the minimum variance controller, the benchmark value of the most widely used minimum variance index (Harris index) can be obtained.

Using the minimum variance index to evaluate non-Gaussian control systems performance may lead to the wrong result [5]. Compared with the minimum variance, the output entropy of the system could be expressed as:

(9) $$H\left(y_{t}\right)=H\left(Fv_{t}+Lv_{t-\tau }\right)$$

*F* is independent of the operating variables; we call it the constant feedback entropy, which can be obtained using the Diophantine equation, and *L* is dependent on the controller. With proper chosen structure and parameters of the controller, *L* = 0, then the entropy of the output reaches the minimum value, which is the minimum entropy (benchmark entropy):

(10) $$H_{\min}\left(y_{t}\right)=H\left(Fv_{t}\right)$$

An entropy-based system performance evaluation index is proposed by Zhang [13]:

(11) $$\eta =\frac{H_{\min}\left(y_{t}\right)}{H\left(y_{t}\right)}$$

where *H_min_*(*y_t_*) is the entropy of the system with the minimum entropy controller, *H*(*y_t_*) is the entropy of the actual output of the system with the current controller. This index has a similar meaning to the minimum variance index, *η* ∈ [0,1]. The closer the value of *η* to 0, the worse the performance of the system. The closer the value of *η* to 1, the better the performance of the control system. 

Shannon entropy cannot meet the consistency requirement and rational entropy is selected for system performance assessment [15]. Rational entropy can meet the consistency requirement, and has the most properties of Shannon entropy:

(12) $$H_{RE}=-\int \gamma (x)\log\frac{\gamma (x)}{1+\gamma (x)}dx,x\in R$$

The entropy has translation invariance, whose value is determined by the shape of the distribution and is independent of the central position of the distribution. For example, two of the same shape distributions with different centers (determined by the mean), the entropies of the two distributions are the same. This leads to inaccurate performance assessment. The PDF of a system error is expected to obey high and sharp distribution at zero, so a constraint to the mean should be added to the system performance assessment with minimum entropy. The performance assessment index that combines minimum entropy with the mean limit is:

(13) $$\eta =\lambda_{1}\eta_{mean}+\lambda_{2}\eta_{me}$$

where *λ*_1_ + *λ*_2_ = 1, *η*_mean_ is the mean index of the system output error, and *η*_me_ is the minimum entropy index. The following *λ*_1_ is chosen to ensure an accurate evaluation of performance when the PDF of the error does not obey the high and sharp distribution at zero:

(14) $$\lambda_{1}=\left|\arctan\left(E\left(e\right)\right)\right|$$

where *E*(*e*) is the mean of system output error *e*; thus, the performance index in Equation (13) is a value between 0 and 1. The closer the value to 1, the closer the system to the ideal case, indicating that the system performs well; otherwise, the performance of the system needs to be improved.

Besides combining the mean with entropy, mixture correntropy can be chosen as the performance assessment index for non-Gaussian control systems to solve the problem of translation invariance.

### 3.2. Correntropy and Mixture Correntropy Index

Correntropy is a measure of similarity in the kernel space and can suppress large outliers in signal processing. The higher the correntropy between two sequences, the smaller the difference between the two sequences. Given two random variables *X* and *Y*, the correntropy between them can be defined as:

(15) $$V(X,Y)=E\left[\kappa (X,Y)\right]=\int \kappa (x,y)dF_{XY}\left(x,y\right)$$

where *E*(.) is the mean, *F_XY_* is the joint PDF of random variables *X* and *Y, x* and *y* are the sampling sequence of random variables *X* and *Y*, respectively, and *κ*(.) is the Mercer kernel. So far, the widely used Mercer kernel function is the Gaussian kernel function. Its expression is:

(16) $$\kappa (X,Y)=G_{\sigma }(e)=\exp(-\frac{e^{2}}{2\sigma^{2}})$$

where *e = x* − *y* and *G*_σ_ (.) is the kernel width of the Gaussian kernel function.

In an actual system, the joint PDF is unknown in most cases, and only a limited amount of data is available. Therefore, the empirical correntropy in Equation (17) is adopted, where *N* is the number of sampling sequences:

(17) $$\overset{\wedge }{V}(X,Y)=\frac{1}{N}\sum_{i=1}^{N}G_{\sigma }(x_{i}-y_{i})$$

The selection of the kernel width of the Gaussian kernel function strongly influences the empirical correntropy. At present, no uniform method is used to select kernel width, but trial and error and the Silverman rule are commonly used.

Mixture correntropy of Gaussian kernel functions with two different kernel widths is proposed by Chen [19]. Mixture correntropy can flexibly adjust the kernel width and weight coefficient to improve the accuracy of the correntropy. For example, when the mixture correntropy is applied in the system identification, the system parameters are more accurate. The mixture correntropy is defined as follows:

(18) $$M(X,Y)=E\left[\alpha G_{\sigma 1}(e)+\beta G_{\sigma 2}(e)\right]$$

where *M*(.) is the mixture correntropy; *σ*1 and *σ*2 are the kernel widths of the Gaussian kernel functions of *G*_σ1_(.) and *G*_σ2_(.), respectively, and α and *β* are weight coefficients of the two kernel functions. For Equation (18), the mixture correntropy can be extended to a generalized form containing a plurality of kernel functions. For simplicity, only two Gaussian kernel functions are considered. Without loss of generality, the kernel width is assumed to be *σ*_1_ ≤ *σ*_2_.

Similar to correntropy, the mixture correntropy is also calculated with an empirical formula as the joint PDF of the actual system is mostly unknown:

(19) $$\overset{\wedge }{M}=\frac{1}{N}\sum_{i=1}^{N}\left[\alpha G_{\sigma 1}(e_{i})+\beta G_{\sigma 2}(e_{i})\right]$$

The mixture correntropy could be considered as a generalized form of correntropy. The expression shows that when one weight coefficient is 0, and the other is 1, the mixture correntropy can be regarded as the correntropy of the kernel function *G*_σ1_(.) or *G_σ2_*(.).

Since the mixture correntropy has more flexibility than the correntropy, with proper chosen weight coefficients, the mixture correntropy can perform better.

Some properties of mixture correntropy are as follows:

Property 1: The mixture correntropy is symmetrical, i.e., *M*(*X*, *Y*) *= M*(*Y*, *X*).
Property 2: The mixture correntropy is positive and bounded, only when *X = Y*, *M*(*X*, *Y*) = 1.

These properties also indicate that mixture correntropy can be used for performance evaluation.

When the expected output of a system is available, the mixture correntropy can be directly used as the index to evaluate the performance of the system. The benchmark mixture correntropy of the system is 1, the mixture correntropy based performance assessment index is:

(20) $$\eta =M(R_{t}-Y_{t})=E\left[\alpha G_{\sigma 1}(e)+\beta G_{\sigma 2}(e)\right]$$

where *R_t_* is the expected output of the system, *Y_t_* is the actual output of the system, and e = *R_t_ − Y_t_*.

When the expected output distribution of a system is unavailable, the mixture correntropy is combined with EDA to get the improved EDA which is given in the following Section 3.3 for system identification; then Equation (11) is chosen as the performance index for the performance evaluation.

### 3.3. System Identification and EDA

According to the feedback control system depicted in Figure 1 and Equations (12) and (13), the PDF of the variable must be known to obtain rational entropy, and the premise of obtaining the PDF is that the order, delay, and parameters of the system model are known.

The simplest method to estimate the delay is to analyze the correlation between the input signal *u*(*t*) and output signal *y*(*t*) [20]. We use the Akaike information criterion (AIC) [21] to obtain the order of the model.

With determined delay and order, the parameter of the system model needs to be identified. The recursive extended least square (RELS) algorithm [22] is used to estimate the system parameters and noise distribution preliminarily, then the mixture correntropy criterion-based EDA is chosen to estimate the parameters of the system and the distribution of noise more accurately. Once the PDF of the noise is obtained, an accurate performance evaluation index is provided.

The EDA [23] is also called the genetic algorithm based on the probability model. In the traditional genetic algorithm, the population is used to represent a set of candidate solutions to the optimization problem. Each individual in the population has corresponding adaptation values. The algorithm performs the operations of selecting, crossing, and variation to simulate natural evolution, and repeats itself to solve the problem. In the EDA, the study and sampling of the probability model are performed instead of traditional genetic operation such as crossover and variation. The EDA describes the distribution of candidate solutions in space using a probability model, establishes a probability model describing the distribution from the macro perspective of the population by means of statistical learning, and then randomly samples the probability model to produce a new population, so that the evolution of the population is realized until the termination condition is achieved.

Crossover and mutation in the genetic algorithm can destroy the individuals that have been optimized. The EDA replaces the crossover and mutation operators in the genetic algorithm with the operations of establishing a probability model and sampling and solves this genetic algorithm problem with a kind of operation mode with global manipulation. The EDA does not require too many parameter settings, and programming is simpler than that of the genetic algorithm.

The EDA is simple in theory, but heavy in calculation burden. Based on the traditional EDA, the initial population estimation is added in this paper, and the improved algorithm learns from the cross-operation of the genetic algorithm. With this improvement, the algorithm can make full use of the best information retained and improve the search speed and optimization accuracy. The improved crossover method is:

(21) $$o^{new}=ao_{\min}+(1-a)o^{old}$$

where *a* is a random number in [0,1] and *o^new^* is the new individual, *o_min_* is the best individual, and *o^old^* is the last sampled individual. The steps of the algorithm could be summarized as follows:

(1) Preliminary estimation of parameters; select the initial population.
  The parameters are roughly estimated by the RELS algorithms and used as the central value of the initial population of the EDA. The parameter space initialization is completed.
(2) Calculate the fitness.
  R individuals are randomly selected from the parameter space, and the corresponding mixture correntropies are calculated. When the difference between the two adjacent mixture correntropies is less than a very small pre-specified value, the cycle ends.
(3) Establish a parameter probability model.
  Select *N* individuals with better fitness in *R*, calculate their means and variances and determine the probability model of the parameters.
(4) Population sampling.
  The parameter population is sampled by the established probability model.
(5) Data intersection.
  Cross some of the data of the population in step (4).
(6) Return to step (2) until the stop criterion is met.

**Figure 2:**
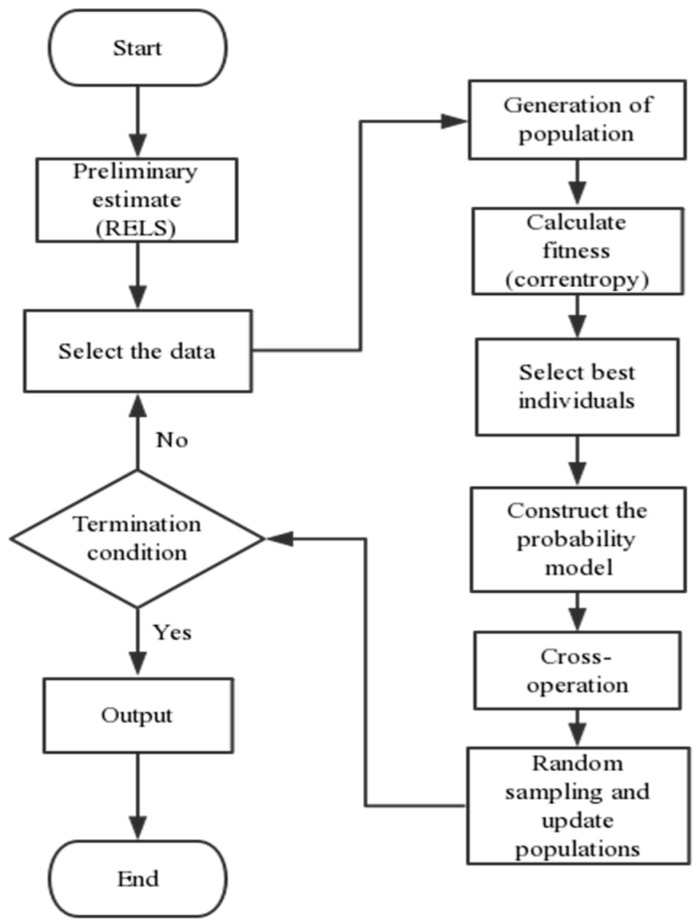
Process of the algorithm.

Figure 2 shows the process of the algorithm.

As the mixture correntropy is combined in the improved EDA, the translation invariance can be avoided. After obtaining the corresponding PDF, the system parameters and noise estimation distribution are obtained, the benchmark entropy and the performance of the system can be calculated according to Equations (10) and (11). Due to the simple calculation of the mixture correntropy, the system performance assessment index could be obtained quickly.

## 4. Selection of System Performance Assessment Indices

When the minimum entropy is used to assess the performance of a system, the expected output distribution of the system is not involved. Therefore, in the case that the expected output distribution is unavailable, the system performance could be assessed with the index in Equation (11) and the mixture correntropy combined with EDA increases the accuracy of the evaluation index.

For a system with available expected output distribution, the ratio between the actual output statistics and the expected output statistics could be used to evaluate the performance of the system. Therefore, when the expected output distribution of a system is known, two simpler and faster indices are adopted:

1. The area of coincidence between the PDFs of the actual output distribution and the expected output distribution, is given in Equation (22).
2. The mixture correntropy-based performance assessment index is given in Equation (20).

To prove the effectiveness and correctness of the performance assessment index based on the mixture correntropy, the performance index based on the PDF coincidence area is briefly introduced in this paper.

**Figure 3:**
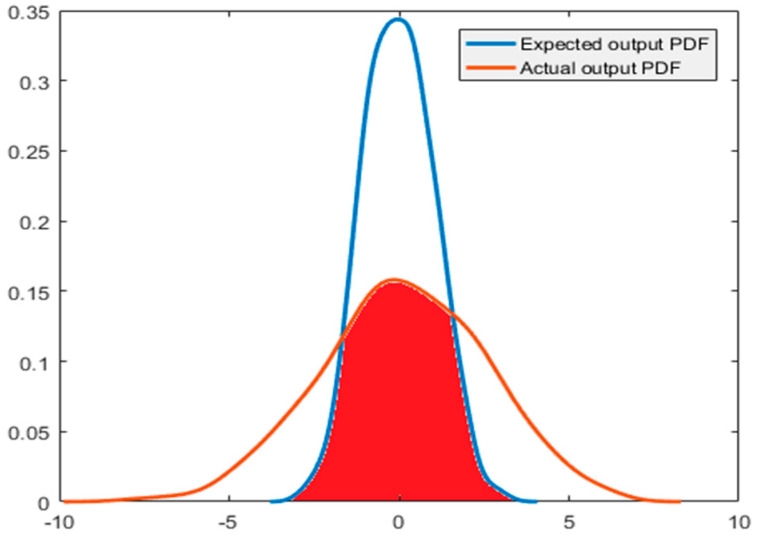
PDF overlap area.

As shown in Figure 3, for a system with a known expected output, the coincident area in the actual output PDF and the expected output PDF can be used as the index of system performance evaluation, which is the red area in the figure. The global integral of PDF is 1; therefore, the result of this performance evaluation index is equal to the value of the coincident area between the actual output PDF and the expected output PDF. That is:

(22) $$\eta =\frac{S_{\mathrm{coincident}}}{S_{yr}}=S_{\mathrm{coincident}}$$

where *S_coincident_* is the coincident area of the two PDF curves, *S_yr_* is the expected output PDF area of the system, which is equal to 1. Therefore, Equation (22) could accurately and rapidly provide the system performance and can be used to prove the effectiveness and accuracy of the mixture correntropy performance assessment index in Equation (20).

## 5. System Simulation

To verify the above identification algorithm and performance evaluation index, the following system is chosen, and the Gaussian and non-Gaussian noise signals are numerically simulated.

(23) $$y(t)=u(t-2)+\frac{1-0.2z^{-1}}{1-z^{-1}}v(t).$$

The transfer function of the controller is

(24) $$G_{c}=\frac{K}{1-0.2z^{-1}-0.8z^{-2}}$$

From the given system, the parameter of the system is *θ*= [−1,1,−1, −0.2], and the delay is *τ* = 2. By solving the Diophantine equation, the feedback invariant *F* = [1,0.8] could be obtained.

**Figure 4:**
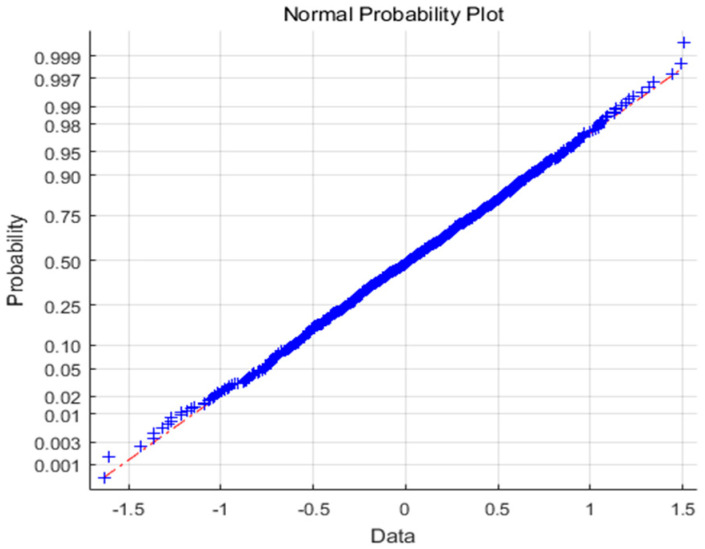
Normal distribution.

**Figure 5:**
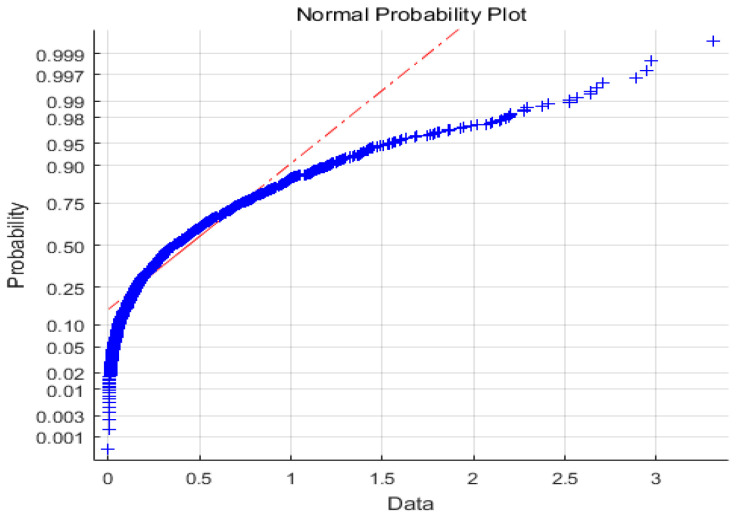
Exponential distribution.

In the simulation, the controller gains *K* = 1.2, assuming that the noise distributions are normal *N*(0,0.255) and the exponential one *E*(0.5). According to Figure 4 and Figure 5, *N*(0,0.255) obeys Gaussian distribution, whereas *E*(0.5) obeys non-Gaussian distribution.

### 5.1. Simulation When Expected Output Distribution is Unavailable

When the expected output of the system is unavailable, the key point for evaluating the performance of the non-Gaussian control system is to obtain the benchmark entropy, and the noise distribution will be used in calculating the benchmark entropy.

In the EDA, the initial population is 1000 groups of data, and the probability model of the parameters is established. For the minimum entropy performance index, the termination condition of the cycle is that the difference between two adjacent rational entropies is less than 0.001. For the performance index of mixture correntropy, the cycle termination condition is that the difference between two adjacent mixture correntropies is less than 0.0001, or the mixture correntropy reaches the maximum value 1.

To improve the accuracy and speed of the algorithm, the RELS algorithm is chosen to determine the approximated range of the initial population. The following Algorithm 1 is the program steps for performance evaluation.

**Algorithm 1** Program steps for the performance evaluation1: Use the CARMA model to represent the system; estimate the delay *τ* by analyzing the correlation between *u*(*t*) and *y*(*t*); use the Akaike information criterion to obtain the order of the model (*n_a_, n_b_, n_c_*).2: *a_n_*, *b_n_*, and *c_n_* in Equation (2) and the estimation of noise variance *σ_v_* are obtained by combining data in step **1** with RELS.3: Take X = [*a_n_* ± 2*σ_v_,*b_n_* ± 2*σ_v_,*c_n_* ± 2*σ_v_] as the initial population of EDA.4: **For i = 1:L** (The value of L is determined by the number of rows of matrix X).  Calculate the mixture correntropy (Equation (19)) of each group of step **3**    **If** M = 1 or the difference between two adjacent rational entropies is less than 0.001, end the cycle.    **Else**    (1) Calculate the estimated average value of the selected N parameters (corresponding mixture correntropy is higher) and establish the probability models.    (2) The parameter population X is sampled by the established probability model.    (3) Population crossover by Equation (21).    **End**
  **End**
5: The optimal estimate of the system parameters and the noise estimated distribution can be obtained by step 4.6: The reference entropy can be obtained by Equation (10).The entropy of output is obtained according to Equation (12).Finally, the results of the performance evaluation index are obtained by Equation (11).

Through the EDA, the system parameters and noise estimation distribution are obtained. The benchmark value of the performance indicator can be calculated according to Equation (11), and the final performance indicator is obtained by dividing the entropy of the actual output.

**Figure 6:**
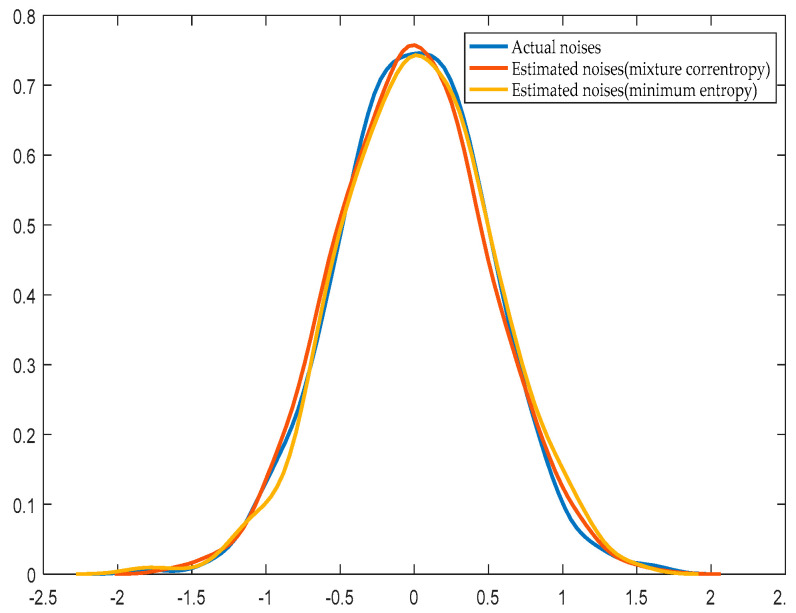
Actual distribution and estimated distribution of noise whose distribution is normal.

**Figure 7:**
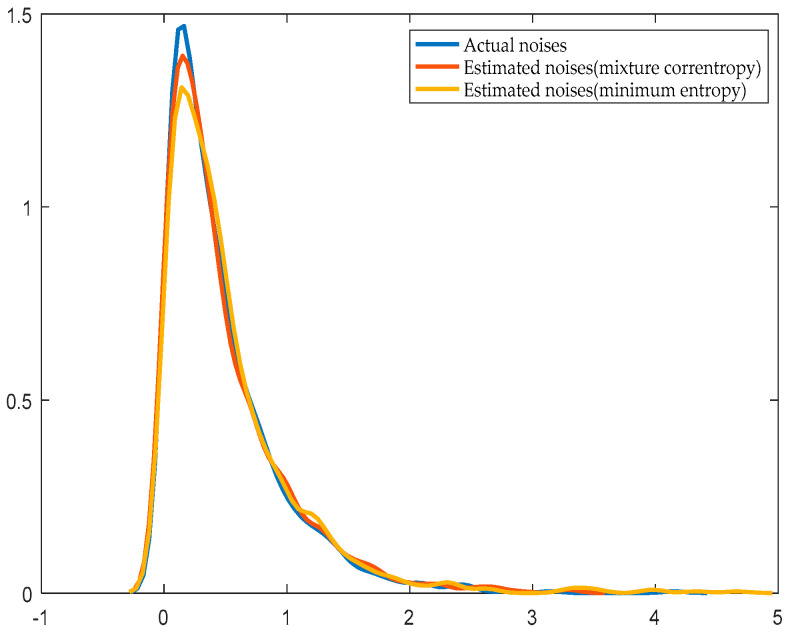
Actual distribution and estimated distribution of noise whose distribution is exponential.

Figure 6 and Figure 7 show the distribution of the estimated noise and the actual noise curve when the noise obeys normal distribution and exponential distribution, respectively.

Compared with the prior system identification through the minimum entropy algorithm, the system parameter identification is more accurate with the improved EDA when the value of the mixture correntropy is larger. The more accurate the system parameter identification, the closer the noise estimation distribution to the actual distribution. The calculation speed is fast, and the iteration process of the improved EDA is faster than that of the prior system identification using the minimum entropy algorithm. Table 1 shows that when the disturbance is subject to a normal distribution and exponential distribution, the identification of the mixture correntropy produces the same or even more accurate results than the identification of the minimum entropy. The time required to obtain the evaluation index is also short. When the number of data increases, the required time clearly increases. The maximum value of the PDF of the error is fixed at *e* = *0* with the index of the mixture correntropy without adding a constraint to the mean.

### 5.2. Simulation When Expected Output Distribution is Available

For the case where the system expected PDF is available, it is assumed that the output of the system obeys a normal distribution with 0 mean and a variance of 1.

The ratios of the coincident area of the PDF and mixture correntropy index all take the form of the Harris index, that is, the ratio of actual value to the expected value related to output. In the previous work, the ratio of the coincident area of output PDF of a system and the PDF of the expected output to the global integral of PDF is used. As the global integral of PDF is 1, the result of this index is equal to the coincident area of the actual output PDF and the desired output PDF.

**Figure 8:**
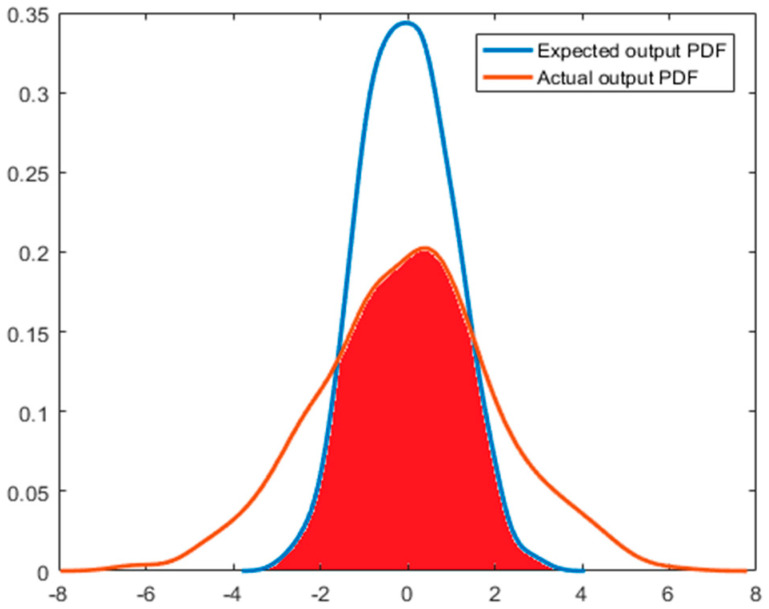
Expected and actual distribution of output of Gaussian systems.

**Figure 9:**
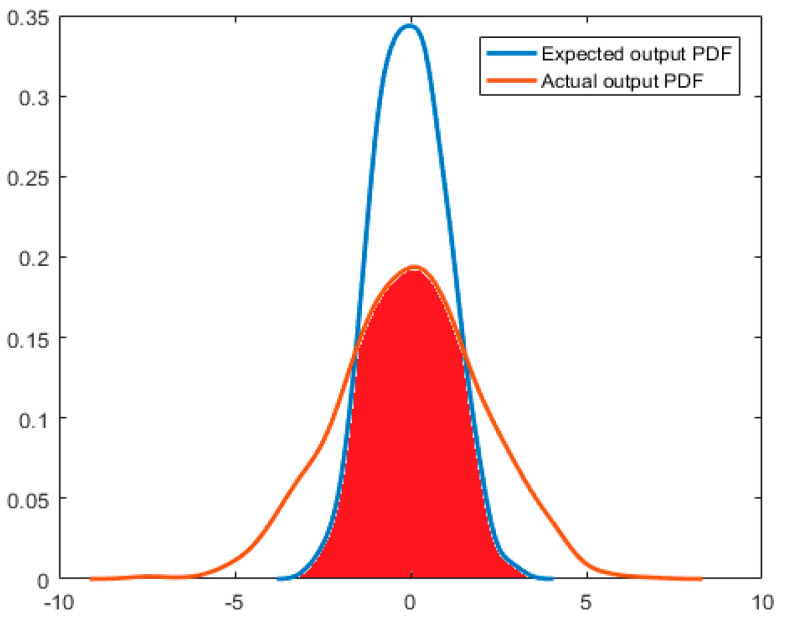
Expected and actual distributions of output of non-Gaussian systems.

Figure 8 and Figure 9 depict the simulation results when the expected output of the system is known. The noise obeys normal distribution in Figure 8, and the noise obeys exponential distribution in Figure 9. Table 2 and Table 3 provide the results of the program running results when the noise obeys Gaussian distribution and non-Gaussian distribution, respectively. According to Table 2 and Table 3, the indexes of the coincident area of the PDF and the index of the mixture correntropy have the same changing trend. Comparing the two indexes with the index of minimum entropy combined with the limit to the mean value, the correctness and rationality of the above two indexes could be improved. In the simulation, we found that less calculation time is needed for the mixture correntropy. When the amount of data is very large, the mixture correntropy index has an advantage in terms of the calculation time, and the results of the system performance assessment could be provided quickly and accurately.

## 6. Summary

To assess the performance of non-Gaussian control systems quickly and accurately, a mixture of correntropy-based performance assessment index and an improved EDA are proposed in this paper. A control system is given first, then with a brief review to the existing performance assessment indices, the correntropy and mixture correntropy are introduced, and a mixture correntropy based performance assessment index is proposed. To assess the distribution of non-Gaussian systems with unavailable expected distribution, the improved EDA is given in detail. The rules for choosing a performance assessment index are given for non-Gaussian systems with the available and unavailable expected distribution. A numerical example is given for the simulation study to compare the proposed performance assessment index and improved EDA with the existing index and algorithm. Both Gaussian and non-Gaussian systems with available and unavailable expected distribution are discussed. The simulation results support the effectiveness and the advantages of the proposed performance index and the improved EDA.

## Figures and Tables

**Table 1:** Parameter estimation and performance evaluation index.

Project	Actual Parameter Value	Gaussian		Exponential	
		Results of Minimum Entropy Identification Method	Results of Mixture Correntropy Identification Method	Results of Minimum Entropy Identification Method	Results of Mixture Correntropy Identification Method
a1	−1	−0.8905	−0.9834	−0.7253	−0.9937
b0	1	1.0054	1.0566	0.9178	0.9634
b1	−1	−1.0051	−0.9432	−0.9191	−0.9691
c1	−0.2	−0.1871	−0.1952	−0.2652	−0.2907
*mixture correntropy*			0.9985		0.9928
*evaluation index*		0.8347	0.8733	0.8202	0.8513
*Time required (s)*		16.7529	11.0601	15.9768	10.8152

**Table 2:** Three performance indices for the Gaussian system with available expected distribution.

Index	Mixture Correntropy Performance Index	PDF Coincidence Area Performance Index	Mean-Limited Minimum Entropy Index
1	0.5752	0.5090	0.7232
2	0.5848	0.5171	0.7385
3	0.6137	0.5288	0.7441

**Table 3:** Three performance indices for non-Gaussian system with available expected distribution.

Index	Mixture Correntropy Performance Index	PDF Coincidence Area Performance Index	Mean-Limited Minimum Entropy Index
1	0.5611	0.5334	0.7458
2	0.5937	0.5516	0.7577
3	0.6127	0.5842	0.7701
